# Patient-Derived Organoids: The Beginning of a New Era in Ovarian Cancer Disease Modeling and Drug Sensitivity Testing

**DOI:** 10.3390/biomedicines11010001

**Published:** 2022-12-20

**Authors:** Iason Psilopatis, Alexandros G. Sykaras, Georgios Mandrakis, Kleio Vrettou, Stamatios Theocharis

**Affiliations:** 1 First Department of Pathology, Medical School, National and Kapodistrian University of Athens, 75 Mikras Asias Street, Bld 10, Goudi, 11527 Athens, Greece; 2 Department of Gynecology, Charité—Universitätsmedizin Berlin, Corporate Member of Freie Universität Berlin and Humboldt—Universität zu Berlin, Augustenburger Platz 1, 13353 Berlin, Germany; 3 Department of Cytopathology, Aretaieion Hospital, Medical School, National and Kapodistrian University of Athens, 11528 Athens, Greece

**Keywords:** organoids, ovarian cancer, three-dimensional cell culture

## Abstract

Ovarian cancer (OC) is the leading cause of death from gynecological malignancies. Despite great advances in treatment strategies, therapeutic resistance and the gap between preclinical data and actual clinical efficacy justify the necessity of developing novel models for investigating OC. Organoids represent revolutionary three-dimensional cell culture models, deriving from stem cells and reflecting the primary tissue’s biology and pathology. The aim of the current review is to study the current status of mouse- and patient-derived organoids, as well as their potential to model carcinogenesis and perform drug screenings for OC. Herein, we describe the role of organoids in the assessment of high-grade serous OC (HGSOC) cells-of-origin, illustrate their use as promising preclinical OC models and highlight the advantages of organoid technology in terms of disease modelling and drug sensitivity testing.

## 1. Introduction

Ovarian cancer (OC) represents the most common cause of death from malignancies of the female genital tract and the fifth most common tumor-related cause of death in women in the United States [1]. According to the American Cancer Society, about 19,880 new cases of OC will be diagnosed and about 12,810 women will die from OC in the United States in 2022 [1]. There are several different histological types of OC. Epithelial carcinomas comprise the vast majority of OCs, with high-grade serous OC (HGSOC) representing the leading morphological subtype [2]. OC is typically diagnosed in advanced tumor stages, and after metastasis has already occurred, given the lack of specific symptoms of the disease at its early stages [3]. As a consequence, the 5-year survival rate for women diagnosed with invasive epithelial OC drops to 31% when a distant metastasis is present [4]. Diagnostic evaluation of OC includes, in addition to a physical examination, a transvaginal ultrasound (TVUS) and a Computed Tomography (CT) scan of the abdomen/pelvis, eventually combined with a Positron Emission Tomography (PET) scan. Definite diagnosis requires histologic examination of the tumor mass, commonly conducted after removing the tumor through surgery [5]. The CA-125 blood test might not be suitable as a screening test for OC, but CA-125 represents a useful tumor marker for the evaluation of treatment efficacy in women with OC [6]. For patients with early-stage resectable OC, hysterectomy with bilateral salpingo-oophorectomy represents first-line therapy. Patients with more advanced stage OC may be also treated with adjuvant chemotherapy. The most commonly used combination includes carboplatin (or cisplatin) and a taxane, whereas the Vascular Endothelial Growth Factor (VEGF) inhibitor bevacizumab might be given along with chemotherapy as well [7].

The generation of reliable pre-clinical models for the study of OC is critical for the development of personalized therapies and is a quest of imperative importance. The ideal pre-clinical model should be reproducible and should fully recapitulate the biological and morphological characteristics of OC. Several pre-clinical OC experimental models have been developed up until now, including 2D cell lines, patient-derived xenografts (PDX), patient-derived explants (PDE), animal models (genetically engineered mouse models-GEMMs) and patient-derived organoids (PDO) [8,9]. OC cell lines are the simplest, most affordable and the most common model used for OC research. Dozens of OC cell lines have been generated and they can be classified in groups representing the different histologic types of OC [10]. However, these cell lines cannot fully reflect the heterogeneity of OC and are not fully characterized on a molecular-genetic level [11]. Moreover, comparative genomic and transcriptomic analyses showed little correlation between OC cell lines and OC clinical samples of the same histologic type and the extensive passaging of cell lines may introduce genetic alterations with unpredictable biological significance [12]. Although they are invaluable surrogate tools for basic research and drug screening, it is unclear to what extent data generated from cell line research are clinically meaningful.

The principle of PDX development is the transplantation of fresh OC tissue into immunodeficient mice. The success rate of OC PDX implementation varies significantly between studies but it universally accepted that the xenografts faithfully recapitulate the histology and the genomic profile of the original cancer tissue [13]. However, the lack of a functional immune system in the recipient mice precludes the use of PDX models as experimental tools for immune-related OC therapies [14]. Moreover, the murine stroma quickly replaces the human stroma, leading to transcriptomic alteration of OC cells and clonal selection [8,15]. Additionally, the generation of PDX models is rather laborious and their genetic manipulation is not easy. On the other hand, PDE models are easy to generate as they represent ex vivo cultures of freshly resected OC tissue [16]. Despite their feasibility and their morphologic and genetic similarity with OC tissue, PDE models have limited use in the study of OC, mainly because of viability problems and their short-term nature that restricts the potential applications of these explants. GEMMs are considered as ideal preclinical models for mechanistic studies regarding cancer development and the crosstalk between OC cells and tumour microenvironments. Although the cellular origin of HGSOC remains debatable, several mouse models have been developed mainly for HGSOC [17] but also for other types of epithelial OC. These GEMMs develop tumours that differ in terms of histological heterogeneity, molecular profile and response to therapy even if they are generated using identical genetic alterations targeting the ovarian surface epithelial (OSE) cells or the fallopian tube epithelial (FTE) cells [18]. Similar to PDX models, GEMMs require the use of animal facilities and are laborious and expensive.

Organoid technology was introduced in 2009 as a groundbreaking 3D primary tissue culture model and was quickly developed as a sophisticated and promising preclinical model for cancer research [19,20]. Organoids are self-organized 3D-tissue structures that reflect the biology and/or pathology of the tissue they are derived from [21]. Organoids are derived from stem cells (pluripotent or tissue-resident) or differentiated normal or cancer cells and they mimic the biological and the functional profile of healthy or cancer tissues [22]. It is not surprising that organoids have piqued the interest of many researchers in oncology as they enable us to reconstruct normal or abnormal tissues in vitro, recapitulate their features and manipulate them easily [22,23]. Tumour organoids have been derived from biopsies (tissue or liquid) and resected specimens of various cancers (gastrointestinal, pancreatic, liver, prostate, etc.) and have been used for personalized medicinal applications [22].

Tumour-derived organoids are generated from tumour tissue that undergoes dissociation resulting in single cells or cell aggregates that are seeded in a saturated medium consisting of hormones, nutrients, appropriate growth factors for the desired organ and a fundamental extracellular matrix (ECM), sold by the brand name Matrigel [24]. Ovarian organoids are complex structures mainly because of monthly changes due to the menstrual cycle. The ovaries perform two key functions of predominant importance for reproduction. First, they produce mature oocytes that are released into the fallopian tube and second, they perform an endocrine function by producing sex hormones, namely estrogen and progesterone, the concentrations of which change monthly, leading to repair of OSE after the ovulation. OSE comprises of a single cuboidal epithelium and is characterized by the expression of both epithelial (KRT7, KRT8, KRT18 and KRT19) and mesenchymal (CDH2 and vimentin) cells, which are vital for the plasticity and post-ovulation homeostasis in vivo of OSE by undergoing bidirectional epithelial-mesenchymal conversion [25]. It is therefore well-understood that the ovaries are highly dynamic organs, and their distinguishing self-renewal must be reflected in the organoids in order to accomplish their proper study, both in normal and pathological conditions. Furthermore, patient-derived ovarian organoids engage on different phenotypes as they recapitulate the morphological heterogeneity of patient’s tissue [26]. Additionally, the development of OC organoids is determined by the ECM structure, and it is well-proven that cell survival, oocyte maturation, follicle development, and sex hormone production depend on the collagen density and elasticity [27].

In general, the establishment of ovary organoids follows a similar strategy to that of other organs. According to a common preparation strategy, tissue fragments are first collected from the patient, then shattered into constituent cells by mechanical or enzymatic digestion, which involves incubation in a collagenase solution at 37 °C with continuous stirring for 1–2 h, and finally the tumor cells are cultured in a specific medium containing 75% Matrigel/25% culturing medium [28]. This culturing medium differs in the ingredients and concentrations of certain substances made for each experiment/study but mainly an advanced DMEM/F12 (advanced Dulbecco’s modified Eagle medium/Nutrient Mixture F-12) medium is widely used [28,29,30,31,32,33]. DMEM and F12 are often mixed to provide higher concentrations of DMEM’s components and a wider range of ingredients in Nutrient Mixture F-12. However, DMEM/F-12 contains no growth factors, hormones or lipids; therefore, the necessary combination of proteins-peptides-hormones for growth should be added to the medium. The cocktail of growth factors used for OC organoids propagation is not standard and may differ significantly between studies. In the case of OC organoids, R-Spondin 1 (Wnt pathway activator), Noggin (BMP-dependent differentiation inhibitor) and 17-β Estradiol seem to be required for growth, whereas Neuregulin-1 (NRG1) may be required for organoids’ expansion [26,34]. Although OC organoids cultures have been successfully established, the derivation efficiency of organoids from OC tissue is not consistent (differs from 30% to 90% for HGSOC organoids) and it needs to be improved, in general [8]. The efficiency of OC organoids development depends mainly on the histological type and grade of OC, but also on the biopsy specimen characteristics (tumor abundance, heterogeneity, necrosis, presence of stroma) and on the experimental protocol [35]. OC organoid preparation protocols do not have only different efficiencies; they also differ in terms of the timescale of organoid formation (from one to three weeks) and expansion (from short-term cultures and two passages to long-term cultures of over a year and more than thirty passages) [8,9].

## 2. The Role of Organoids in the Assessment of HGSOC Origins

HGSOC is inconclusively believed to arise from ovarian surface epithelial or fallopian tube secretory cells [36]. Several study groups have attempted to identify the tissue of origin by developing organoids from healthy fallopian tubes and OSE cells. Kessler et al. established long-term, stable organoid cultures from human fallopian tube single epithelial stem cells. Microarray analysis highlighted the significance of Wnt and Notch paracrine signaling pathways for continuous organoid growth and differentiation, and revealed the physiological response of organoids to estradiol and progesterone treatment, thus opening up novel possibilities for studying the etiology of OC [37]. The same study group established 15 organoid lines from HGSOC primary tumor deposits and found that the Wnt pathway activation induces growth arrest, whereas the generation of HGSOC organoids almost always necessitates active Bone Morphogenetic Protein (BMP) signaling. Healthy fallopian tube organoids, on the other hand, were shown to depend on BMP suppression by Noggin, whereas stable short hairpin RNA (shRNA) knockdown of p53, Phosphatase and Tensin homolog (PTEN) and retinoblastoma protein (RB) failed to induce a direct growth advantage of the altered cells in the absence of a low-Wnt environment, thus underlining the importance of early changes in the stem cell niche environment for the outgrowth of genetically altered cells [31]. With a view to investigating the tissue of origin, Lõhmussaar et al. created HGSOC organoid-based tumor progression models from fallopian tube and ovarian surface epithelium tissues. By employing CRISPR-Cas9 genome editing, the Dutch study group demonstrated that both tissues have the ability to give rise to HGSOC and express Paired box protein 8 (Pax8), a previously thought specific oviductal secretory cell marker. Nevertheless, the mutant clones from these tissues showed differential genomic stability and changes in proliferation and apoptosis upon acquiring more mutations, with fallopian-tube-derived tumors outperforming the ovarian-surface-epithelium-derived tumors by greater tumor-derivation, higher proliferation rate or the ability to survive as orthotopic tumors in the bursal environment. Of note, in vitro drug testing revealed a distinct lineage-specific response to paclitaxel and niraparib, two widely used anti-HGSOC agents [38]. Similarly, Zhang et al. developed genetically engineered fallopian-tube- and ovarian-surface-epithelium-derived organoids to investigate the tumor-forming properties of these epithelial cells harboring the same oncogenic modifications. HGSOC was proven to originate from either cell type, yet fallopian-tube-epithelium-derived HGSOC differed in latency, metastatic potential, transcriptome, chemotherapy response and eventually causative genomic alterations from ovarian-surface-epithelium-derived HGSOC. Combined RB family inactivation and Tp53 mutation in Pax8-expressing fallopian tube epithelial cells caused rapid metastasis to the ovarian surface, whereas mutant fallopian-tube-epithelium-derived organoid orthotopic injection recapitulated HGSOC progression. Combined Tp53 mutation and RB family inactivation in Leucine-rich repeat-containing G-protein coupled receptor 5 (LGR5)-expressing ovarian-surface-epithelium cells and ovarian-surface-epithelium-derived organoids also caused HGSOC, yet with a slower growth rate and longer latency [18]. Maru et al. reported that, even though lentiviral Cre-mediated Trp53 deletion did not promote carcinogenesis in fallopian tube organoids, subsequent suppression of PTEN and simultaneous induction of mutant Phosphatidylinositol-4,5-Bisphosphate 3-Kinase Catalytic Subunit Alpha (PIK3CA) cooperated with p53 loss for the development of HGSOC. Interestingly, Kirsten rat sarcoma virus (KRAS) activation cooperated with a loss of p53 expression for the development of carcinosarcoma, with immunohistochemical examination revealing the possible epithelial–mesenchymal transition (EMT) of fallopian-tube-cell-derived OC cells [39]. Xie et al. established an organoid culture system for mouse fallopian tube epithelial cells and found the fimbria of the fallopian tube to be enriched with organoid-forming fallopian tube epithelial stem cells. Given their close proximity to the ovary, the authors speculated that these organoid-forming cells are possibly exposed to follicular fluid during ovulatory rupture and may even become entrapped in the ovary through the rupture site, thus rendering them suspects possibly responsible for ovarian carcinogenesis [40]. Yucer et al. established induced-pluripotent-stem-cells-derived fallopian tube organoids, with a view to creating a trustworthy in vitro OC model, able to recapitulate early de novo genetic alterations, model HGSOC pathogenesis and progression as well as uncover novel therapeutic possibilities [41].

The role of organoids in the assessment of HGSOC origins is depicted in Table 1.

## 3. Efficient Use of Organoids as a Preclinical Model for OC

Organoid culture has become common for patient-derived samples in OC. Chan et al. employed cell viability and cancer organoid assays in order to investigate the effect of overexpression of short-form thymic stromal lymphopoietin (sfTSLP), an epithelial cell derived cytokine, on tumor growth in vitro, and showed significantly higher numbers of viable cells of sfTSLP-expressing ovarian A2780 and IGROV-1 cancer cells, thus confirming its upregulation in OC [42]. Chen et al. cultured organoids from HGSOC malignant effusions and, after performing RNA-sequence analysis of four patient specimens, observed significant upregulation of genes related to cell proliferation, EMT and KRAS signaling pathways, suggestive of transcriptional programs consistent with the proliferative phenotype [43]. Kopper et al. described an organoid platform enabling long-term in vitro expansion, manipulation and analysis of different OC subtypes, and demonstrated that OC organoids not only maintain nuclear and cellular atypia or biomarker expression, but also recapitulate OC recurrent mutations and tumor heterogeneity. Interestingly, unsupervised hierarchical clustering of gene expression data grouped two organoid lines from patients at high risk of developing HGSOC together, thus leading the authors to the assumption that the establishment and analysis of premalignant organoid lines from prophylactic bilateral salpingo-oophorectomy material might create novel opportunities to study early HGSOC development [26]. By modifying the Matrigel bilayer organoid culture (MBOC) protocol, Maru et al. propagated organoids from five ovarian tumors and succeeded in conducting organoid culture with HGSOC, mucinous OC, endometrioid OC and borderline tumors. The organoids retained both histological and genetic characteristics, as well as intra-tumoral heterogeneity, of the original tumors. Specifically, the stereotypical enrichment of variant allele frequencies of TP53 and PTEN mutations in organoids indicated loss of heterozygosity and a point mutation in each gene as a founder mutation in most OC cells, which were enriched as epithelial cells in organoids. On the contrary, the significantly increased variant allele frequencies of Neurofibromatosis type 1 (NF1) and AT-Rich Interaction Domain 1A (ARID1A) mutations in organoids demonstrated intra-tumoral heterogeneity and/or clonal selection during culture [32]. Yucer et al. generated induced-pluripotent-stem-cell-derived, fallopian tube epithelium organoids from healthy women and OC patients with germline pathogenic BReast CAncer 1 (BRCA1) mutations and found that BRCA1-mutated fallopian tube epithelium organoids showed cellular abnormalities consistent with OC genesis and progression, as well as exhibited an increased production of cancer-specific proteins and survival after xenotransplantation. Importantly, HGSOC-derived organoids demonstrated the greatest pathology, thus rendering them potential predictors of clinical severity prior to disease onset, whereas BRCA1-mutated fallopian tube epithelium organoids represented a trustworthy physiological in vitro model of fallopian tube epithelial lesion generation and early tumorigenesis [44]. Zhang et al. developed a fallopian-tube-epithelium-derived organoid-based platform and showed that both *Tp53*^−/−^; *BRCA1*^−/−^; *Myc*^OE^ and *Tp53*^−/−^; *PTEN*^−/−^; *NF1*^−/−^ fallopian-tube-epithelium-derived organoids cause HGSOC-like tumors. Moreover, *AKT2* and/or *KRAS* were found to cooperate with Cyclin E1 (CCNE1) to give rise to HGSOC, whereas organoid genotype influenced genome stability, drug response and secretome, with RNA-sequencing analysis revealing distinct transcriptomes for OC with different genotypes. Of note, tumorigenic organoids evoked distinct immune microenvironments which could be regulated by neutralizing organoid-produced chemo-/cytokines [45]. Very recently, OC organoid cultures were established from induced cancer initiating cell (iCICs). These cells were generated from the HGSOC cell line OVCAR-3 after reprogramming with the Yamanaka factors OCT4, SOX2, KLF4 and MYC (OSKM). The iOVCAR-3-OSKM organoids recapitulate successfully the histopathology of OC and may represent a valuable model for the study of OC pathogenesis [46]. OC organoids are not only prepared from primary or metastatic tumor, but also from body fluids containing OC cells. The growth of ascites-derived OC organoids is attenuated by extracellular vesicles isolated from malignant ascites (MA-EVs). MA-EVs stimulate a significantly larger expansion of organoids than extracellular vesicles derived from benign ascites [47]. Collectively, these studies highlight that OC organoids recapitulate the histological and genetic features of OC, regardless of the preparation method that differs between studies.

The use of organoids as a preclinical model for OC is summarized in Table 2.

## 4. Organoid Culture of OC for Disease Modeling and Drug Sensitivity Testing

Systemic platinum-taxanes combination chemotherapy represents the standard first-line chemotherapy for OC patients [48]. Nevertheless, recurrence occurs in more than 80% of patients with advanced OC because of chemotherapy resistance [49]. Many study groups have therefore attempted to develop OC organoids aiming at identifying possible relevant mechanisms and discovering novel and effective therapeutic alternatives.

Bi et al. performed drug sensitivity assays on 19 OC and endometrial cancer patient-derived organoids and concluded that taxanes seem to be the predominant driver of therapeutic effectiveness in the systemic platinum-taxanes combination chemotherapy. Moreover, second-line therapeutic regimens including bevacizumab, gemcitabine or topotecan were not superior to first-line chemotherapy, whereas drug response testing reflected resistance to different agents given in the neoadjuvant setting [50]. Chen et al. used short duration organoid cultures from HGSOC malignant effusions as a platform for empiric drug response testing and demonstrated that the small-molecule p53 reactivator eprenetapopt, the inhibitor of p97/valosin-containing protein (VCP) CB-5083, the small-molecule WEE1 inhibitor adavosertib (MK-1775) and the multiple tyrosine kinase inhibitor (TKI) sorafenib showed the most profound inhibitory effects, with CB-5083 and MK-1775 exhibiting consistent growth inhibitor effects in low micromolar ranges in all organoids [43]. DeathPro represents an automated microscopy-based assay to screen cells from OC organoids with clinically relevant agents. Using DeathPro, Jabs et al. suggested that the histone deacetylase (HDAC) inhibitor belinostat, the phosphatidylinositol 3-kinase (PI3K) kinase inhibitor BKM120 and carboplatin represent the most effective therapeutic agents and that the efficacy of the TKI dasatinib, the mammalian target of rapamycin (mTOR) inhibitor temsirolimus and the mTOR inhibitor AZD2014 depend on OC culture type [51]. Phan et al. established patient-derived HGSOC and ovarian carcinosarcoma organoids and used an automated screening platform to identify individual responses of organoids after exposure to 240 kinase inhibitors. BGT226 showed activity in all tumors, whereas different organoids were found to exhibit differential responses to agents targeting the same pathways [52]. De Witte et al. employed 36 patient-derived OC organoids for ex vivo drug screening and observed low responsiveness to carboplatin/paclitaxel, PARP inhibitors, the TKI afatinib and adavosertib, and high responsiveness to gemcitabine, the Cyclin-Dependent Kinase (CDK) inhibitor flavopiridol and the BRAF V600E kinase inhibitor vemurafenib [53]. Of note, given that HGSOC usually has mitogen-activated protein kinase (MEK) pathway activation but a lack of BRAF mutation, MEK inhibition is currently actively under investigation in HGSOC, with Cappuccio et al. reporting the first case of recurrent HGSOC with profound clinical, radiologic and biochemical response to the MEK inhibitor trametinib [54], and Chesnokov et al. describing in vitro cell cycle arrest in G1/0-phase and in vivo HGSOC growth inhibition following trametinib treatment in cisplatin-resistant cells [55].

D’Amora et al. used ovarian- and uterine-adenocarcinoma-derived organoids for the measurement of individual patient platinum resistance, ex vivo. Two thirds of the 47 included patients achieved complete remission with a mean progression time of almost two years, disease-free survival (DFS) of 1.7 years and overall survival (OS) of 2.6 years. Mean cisplatin lethal concentration 50% (LC50) was associated with a non-significant decrease in complete remission, reduced DFS as well as biochemical signatures of numerous metabolites. Interestingly, receiver operating curves (ROC) of lipid ratios, branched chain amino acids and the tryptophan to kynurenine ratio represented highly sensitive and specific tools for the identification of patients at the highest risk of relapse and death [56]. Gorski et al. utilized six HGSOC tumor organoid lines to screen for carboplatin sensitivity at different doses and found the organoid line UK1254 to be resistant to carboplatin and have a significantly shorter progression-free survival (PFS), with subsequent gene expression analysis identifying the interplay between various pathways related to nuclear factor kappa B (NFκB), PRDM6 or Phosphoinositide-3-Kinase Adaptor Protein 1 (PI3KAP1) activation [57]. By employing and xenografting OC organoids for (in vivo) drug-screening assays, Kopper et al. proved most HGSOC organoids to be sensitive to platinum-based chemotherapy, whereas low-grade serous OC, mucinous and borderline tumors showed increased chemotherapy-resistance. Notably, after comparing chemotherapy responses in matched organoid lines derived from primary chemosensitive and recurrent chemoresistant HGSOC of a single patient, an increased resistance of the organoid line derived from the recurrent HGSOC to platinum-based chemotherapy could be confirmed [26].

McCorkle et al. analyzed paclitaxel resistance in patient-derived OC organoids and found elevated ATP-binding cassette subfamily B, member 1 (ABCB1) expression to correlate with chemoresistance in the organoid lines [58]. Organoids have been used for drug screening in cases of chemoresistance. CWP232291, a small molecule that targets the Wnt/β-catenin pathway by inhibiting the transcriptional activity mediated by b-catenin, has an inhibitory effect on OC organoids from both cisplatin-sensitive and cisplatin-resistant patients [59].

In order to assess HGSOC chemoresistance in a tissue-similar environment, ascites-derived HGSOC cells from women subjected to either primary debulking surgery or neoadjuvant chemotherapy treatment were grown into short-term organoids. Pietilä et al. suggested that collagen-6 adhesion was upregulated by cisplatin, and that collagen-6 enhanced protection against cisplatin cytotoxicity, especially in relapsed HGSOC oganoids [60]. Sun et al. established organoids using cisplatin-sensitive and -resistant OC tissues. The serine/threonine kinase Aurora-A was found to enhance chemoresistance through suppression of cell senescence and implication of glucose metabolism, with significantly increased levels in cisplatin-resistant organoids [61]. RNA-sequencing of cisplatin-resistant and -sensitive OC organoids revealed enhanced fibrillin-1 (FBN1) expression in platinum-resistant OC organoids that correlated with vascular endothelial growth factor receptor (VEGFR) 2/signal transducer and activator of transcription 2 (STAT2) signaling axis activation and consequent angiogenesis and glycolysis modulation. Inspired by these observations, Wang et al. suggested combination of FBN1-knockout with the antiangiogenic agent apatinib for chemotherapy-sensitivity improvement in OC [62].

An in vitro organoid drug assay, which was employed in order to test the efficacy of carboplatin-ReACp53 synergy in OVCAR3 organoids, indicated the presence of increased apoptosis of OVCAR3 cells upon combination of carboplatin with ReACp53 [63]. A second study group was able to show that ReACp53 rescues p53 function in HGSOC organoids, thus promoting the induction of cell cycle arrest and apoptosis [64].

Singh et al. utilized a three-dimensional organoid bioassay to test the efficacy of the carboplatin-birinapant combination therapy and highlighted its synergistic effects on a subset of platinum-resistant OC, with birinapant representing a potent second mitochondrial activator of caspase (SMAC) mimetic targeting inhibitors of apoptosis [65].

Wambecke et al. developed OC organoids from patients with incomplete response to carboplatin and suggested that inhibition of Ubiquitin Conjugating Enzyme E2 N (UBE2N) sensitized OC cells to platinum-based chemotherapy via proapoptotic B-cell lymphoma 2 (BCL2) family protein BCL-2 Interacting Mediator of cell death (BIM) upregulation [66].

Hill et al. created a platform for functionally profiling DNA repair in short-term patient-derived HGSOC organoids and found a functional defect in homologous recombination to be associated with Poly (ADP-ribose) polymerase (PARP) inhibitor sensitivity, as well as a functional defect in replication fork protection to correlate with carboplatin, and Checkpoint Kinase 1 (CHK1) and Ataxia telangiectasia and Rad3-related protein (ATR) inhibitor sensitivity [30]. By performing a cell viability assay to evaluate drug sensitivity in a patient-derived HGSOC organoid line, Jia et al. proved that the combination of the Reactive Oxygen Species (ROS)-inducing agent Phenethyl isothiocyanate (PEITC) with the PARP inhibitor BMN 673 exerted promising therapeutic effects [67]. Nanki et al. developed expandable patient-derived OC organoids and described that the BRCA1-mutated organoid was more sensitive to both platinum drugs and olaparib, whereas a clear cell OC organoid showed resistance to platinum-based chemotherapy, paclitaxel and olaparib [33]. NPB, the small molecule inhibitor of BADS99 phosphorylation, synergizes with PARPi to reduce cell survival in organoids derived from patients with recurrent OC, demonstrating that the down-regulation of pBADS99 in combination with the effect of PARpi may become a therapeutic strategy in recurrent OC [68]. OC organoids have become valuable tools for functional testing and evaluation of drug response. Recently, it was demonstrated that OC organoids recapitulate the response of OC patients to chemotherapy and show response heterogeneity to PARPi. Not only are they used as experimental tools for PARPi sensitivity studies, but they represent platforms for the mechanistic study of chemoresistance and testing of novel drug combinations that can bypass resistance mechanisms [69].

By performing immunofluorescence imaging of a patient-derived, ovarian carcinosarcoma organoid culture, Bi et al. demonstrated the hyperstaining of p53 protein. Computational modeling highlighted the significance of this residue in terms of protein conformation maintenance, whereas drug sensitivity testing proved the combination of bortezomib with belinostat to represent the most effective treatment [70]. The same study group investigated the mechanisms underlying sensitivity and resistance to the combination of HDAC and proteasome inhibitors and showed that nanomolar concentrations of ixazomib and romidepsin synergistically induce cell death in most patient-derived OC organoid models, with autophagy being the main mediator of cell survival in resistant cells, as evidenced by an enhanced antitumor response both in vitro and in vivo [71]. Qian et al. examined the in vitro activity of the HDAC inhibitor PXD101 in OC organoids and concluded that PXD101, used in combination with either paclitaxel or carboplatin, most effectively enhances growth inhibitory activity [72].

Cao et al. employed short-term HGSOC organoids to validate the effect of tumor infiltrating mast cells on anti-PD1 therapy and described that organoids derived from stromal-tumor-infiltrating-mast-cells-low women were correlated with a better response to anti-PD-1 treatment [73]. Wan et al. performed immune functional and single cell RNA-sequencing transcriptional profiling on HGSOC organoid/immune cell co-cultures and noted that treatment with the bispecific anti-PD-1/PD-L1 antibody resulted in natural killer (NK) and T cell induction to more active and cytotoxic phenotypes, through downregulation of the bromodomain-containing protein 1 (BRD1) [74]. Additionally, Zhang et al. reported on the development of a therapeutic regimen combining gemcitabine, granulocytic myeloid-derived suppressor cells (g-MDSCs) as well as anti-PD-L1 antibodies, which yielded durable, T-cell dependent responses in *Tp53*^−/−^; *Ccne1*^OE^; *Akt2*^OE^; *Kras*^OE^ HGSOC tumors [45].

In order to appreciate the cytotoxic effects of the Naftopidil combination with ABT-737 or Trametinib, Florent et al. established three HGSOC organoid lines and, after performing cell viability assays, confirmed that, even though Naftopidil, ABT-737 or Trametinib used alone did not show noticeable effects on organoid morphology, the combination of Naftopidil with ABT-737 disintegrated the organoid structure [75].

Liu et al. investigated the antitumor effects of Stichoposide C and found that this triterpene glycoside succeeded in inhibiting the growth of two patient-derived OC organoids [76].

The CDK4/6 inhibitor Palbociclib showed a synergistic lethal effect on promoting cell cycle arrest and inducing apoptosis in patient-derived OC organoids, once combined with the bromodomain protein 4 inhibitor AZD5153 that inhibits the cell cycle-related protein and mitogen-activated protein kinase (MAPK)/PI3K-protein kinase B (AKT) pathway [77].

McDowell et al. utilized RNA-sequencing analysis to identify genes and the relevant genetic pathways which showed differential regulation in artesunate resistant vs. sensitive OC organoid models, and found G1/S-transition-related pathways to be upregulated in artesunate resistant OC organoids [78].

Unlike carboplatin, Palladium (II)-η^3^-Allyl 4c complex bearing N-trifluoromethyl N-heterocyclic carbenes was very active on patient-derived HGSOC organoids, with low activity on normal liver organoids [79].

By culturing organoids directly from patients with clear cell OC, Shigeta et al. proved the synergistic effect of bromodomain and extra-terminal domain (BET) and PI3K-AKT-mTOR inhibitors on p53-independent apoptosis induction [80].

Vernon et al. investigated the treatment effects of an epidermal growth factor receptor (EGFR) inhibitor in combination with a BH3-mimetic molecule in four patient-derived OC organoids, with cell viability assays revealing synergistic effects of the erlotinib-ABT-737-combination. Notably, these pharmacologic inhibitors mimic the antitumor effect of microRNA-3622b-5p, which inhibits BCL/XL in OC cell lines escaping BIM induction, hence sensitizing them to cisplatin [81].

The use of organoids for disease modeling and drug sensitivity testing is summarized in Table 3 and Table 4.

## 5. Advantages and Limitations of OC Organoids-Future Directions

OC organoids represent an experimental model that overcomes major limitations of other preclinical models such as 2D cell lines and PDX. OC organoids faithfully recapitulate the histology and pathophysiology of OC subtypes, can undergo expansion and manipulation and are ideal for high-throughput drug screening. Thus, they are considered as unique platforms that bridge the gap between in vitro and in vivo models and hold promise for providing meaningful data that will shed light to OC pathogenesis and drive clinical decisions [82].

Despite the enormous potential, OC organoid technology has several drawbacks that impair a widespread adoption as the principal preclinical model for OC. The first limitation is technical. The OC organoids preparation is time-consuming and expensive (due to the cost of Matrigel) in comparison to cell lines. Moreover, there is no standard protocol for OC organoids establishment, resulting in differences to the efficiency of each method, passaging and timeline of culture as well as in the morphology and functionality of organoids. The second limitation concerns the absence of the tumor microenvironment (TME) since the OC organoids include the epithelial cells but not the stroma, the immune cells and the vasculature present in OC. This limitation partially impairs the functionality and the heterogeneity of the system and may alter the response of organoids to drug screening assays. These two major limitations of the organoid system lead to lack of technical, morphological and functional reproducibility of organoid-based studies and constitute a major bottleneck that we need to overcome [82].

The first step that is required for a broader use of OC organoids is the standardization of the protocols for organoid generation and the extensive characterization and validation of the methods that are currently in use. OC organoids need integration of biochemical and biophysical cues from the ECM and TME to be fully representative of OC tissues. Co-culture systems of ovarian OC with elements of TME (cancer associated fibroblasts, endothelial cells and immune cells) would allow for a better spatio-temporal resolution and the minimization of intra-organoid heterogeneity. In fact, this approach has been already implemented in pilot studies with promising results [8,74]. Moreover, the replacement of Matrigel with synthetic ECM will provide factors for the differentiation and long-term expansion of OC organoids. The organoid-on-a-chip is a cutting edge technology that may solve conventional organoids’ limitations by integrating OC cells with the components of OC TME [83]. Organoids-on-a-chip are novel experimental tools based on microfluidic devices containing organoids in a controlled environment that mimics TME and allows for the controlled perfusion of growth factors enabling cell to cell and cell to stroma interactions [84]. This model is very promising for biobanking and drug screening studies but remains to be validated in OC.

## 6. Conclusions

**Figure 1:**
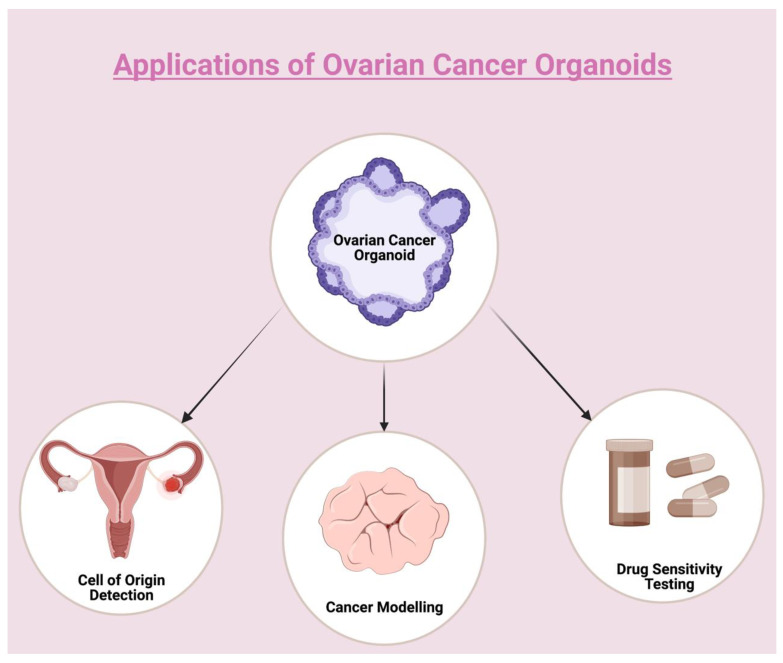
The use of OC organoids for HGSOC cell of origin detection, disease modeling and drug sensitivity testing. Created with BioRender.com.

Even though OC has long been the point of interest of numerous studies, many questions regarding the cells-of-origin, the pathophysiological pathways or the most appropriate therapeutic regimen still remain unanswered. Since the introduction of organoids as self-organizing organotypic structures grown from tissue-derived cells in cancer research, a new era of comprehensive and faithful studies allowing for functional testing, therapeutic sensitivity prediction, and biomarker interrogation has begun [85,86]. Patient-derived HGSOC organoids enable a closer and more reliable study of the dualistic HGSOC origin, as well as the earliest stages of HGSOC development. Furthermore, they represent useful preclinical models for the identification of OC progression-associated mechanisms and clinically-relevant biomarkers. Most importantly, organoids may be applied in drug screening to test the efficacy of first-line chemotherapy, identify therapeutic alternatives for chemo-resistant OC and demonstrate the correlation between genetic mutations and sensitivity to targeted therapies (Figure 1). However, generation and expansion of patient-derived OC organoids with growth medium adaption in accordance with histologic subtypes, as well as integration of the TME incorporating stromal, immune and vascular cells, are necessary in order to recapitulate the molecular features and heterogeneity of the original tumors, thus allowing for the development of comprehensive OC organoid biobanks. In addition, drug screening results have to be compared with clinical outcomes in a standardized manner and examined in large, adequately designed randomized clinical trials, so as to further evaluate the organoids’ capacity to predict the treatment efficacy of chemotherapy and targeted therapy in the clinical setting.

## Figures and Tables

**Table 1:** The role of organoids in the assessment of HGSOC origins.

Organoids	Methods	Main Results	References
**Human-derived organoids**			
Three dimensional organoid cultures from normal human fallopian tubes	Immunohistochemistry (IHC), Microarray expression profiling, Real-time PCR (RT-PCR)	Faithful recapitulation of the mucosal fold architecture by organoids Active Wnt and Notch signaling preserves stemness in fallopian organoids	[37]
Fifteen organoid lines from human peritoneal and omental HGSOC	Flow cytometry (FC), Luminescent cell viability assay, IHC, Immunofluorescence (IF) staining, Quantitative reverse transcription–PCR (qRT-PCR), Western blotting (WB), Drug sensitivity array, Next-generation sequencing (NGS)	HGSOC organoids match tumor tissue in mutational profile and expression of biomarkers	[31]
Human fallopian tube epithelium organoid in vitro model	RT-PCR, IHC, IF	Wnt and BMP signaling modulation directed induced pluripotent stem cell differentiation into Mullerian cells Use of pro-Mullerian growth factors promoted fallopian tube epithelium precursors	[41]
**Mouse-derived organoids**			
Murine fallopian-tube-epithelium-derived and ovarian- surface-epithelium-derived organoids	IF, WB, RNA-sequencing (RNA-seq)	Perturbing Tp53 and the RB family in Pax8-expressing cells cause HGSOC and metastasis Combined Tp53 mutation and RB family inactivation in LGR5-expressing ovarian surface epithelial cells cause HGSOC Transcriptomic and genomic differences between fallopian-tube-epithelium-derived and ovarian-surface-epithelium-derived HGSOC	[18]
Organoid-based tumor progression models of HGSOC from murine fallopian tube and ovarian surface epithelium tissues	Organoid growth assay, FC, IHC, Organoid transfection and genotyping, WB, qRT-PCR, In vitro drug screen, In vivo transplantation assays	Fallopian tube and ovarian surface epithelium organoids show distinct characteristics and differential drug responses Fallopian tube tumors resemble the molecular subtypes of HGSOC	[38]
Murine fallopian tube organoids, tumor-derived organoids	Tumorigenicity assay, WB, Histopathological analysis, Transcriptome analysis, Drug sensitivity assay	Cooperation of the activated PI3K pathway with p53 loss for the development of HGSOC Cooperation of KRAS activation with p53 loss for the development of carcinosarcoma. Diverse carcinogenesis is achieved by genetic cooperation involving KRAS activation, without p53 loss	[39]
Murine healthy fallopian tube organoids	RNA quantification, RT-PCR, IF	Mouse fallopian tube epithelial cells can be cultured as long-term organoids, requiring Notch and Wnt signaling	[40]

**Table 2:** The use of organoids as a preclinical model for OC.

Organoids	Methods	Main Results	References
**Human-derived organoids**			
Cancer organoid formation of A2780 and IGROV-1 human cancer cells with sfTSLP overexpression or empty-vector expression	Tumor Growth Assay	Significantly higher numbers of viable cells of sfTSLP-expressing ovarian A2780 and IGROV-1 cancer cells	[42]
Organoid culture from human HGSOCmalignant effusions	Short-term organoid growth assay, RNA-seq	Recapitulation of the histological features of malignant ascites fluid Significant upregulation of genes related to cellular proliferation, EMT and KRAS signaling pathways	[43]
Fifty-six organoid lines from 32 patients, representing all main subtypes of OC	Scanning electron microscopy, Genomic analysis, RNA-seq, Methylation analysis	Organoids faithfully recapitulate OC at the genomic level and tumor heterogeneity Low-grade serous OC organoids are more similar to normal samples than HGSOC	[26]
Nine human OC-derived organoids	Targeted next- generation sequencing analysis, Cell proliferation assay, Drug sensitivity assay, Tumorigenicity assay	Recapitulation of histological features in organoids Maintenance and enrichment of tumor-derived somatic mutations in organoids Preservation of intra-tumoral heterogeneity in organoids	[32]
Induced-pluripotent-stem-cell-derived, fallopian tube epithelium organoids from healthy women and OC patients with germline pathogenic *BRCA1* mutation	WB, RT-PCR, IHC, Transcriptional Analysis	*BRCA1*-mutated fallopian tube epithelium organoids recapitulate ovarian tumorigenesis and show precancerous pathological changes observed in pre-neoplastic OC lesions	[44]
Organoids generated from human induced OC initiating cells	In vitro differentiation assays	Recapitulation of OC histologic features	[46]
Human ascites-derived OC organoids	Organoid growth assays	MA-EVs induced growth of ascites-derived OC organoids	[47]
**Mouse-derived organoids**			
Murine fallopian-tube- epithelium- derived organoid-based platform	Chemotaxis assays, FC, IF, IHC, WB, RNA-seq, Shallow Whole Genome Sequencing	*AKT2* and/or *KRAS* cooperation with *CCNE1* to cause HGSOC Distinct transcriptomes in OC with different genotypes HGSOC microenvironment dependency on tumor genotype	[45]

**Table 3:** The use of organoids for disease modeling and chemotherapy sensitivity testing.

Therapeutic Agent	Organoids	Main Results	References
**Human-derived organoids**			
Carboplatin	Two neoadjuvant- carboplatin- exposed and four chemo- naïve HGSOC organoid lines from tissue obtained during debulking surgery	UK1254 was predicted to be resistant to carboplatin based on its EC50 value	[57]
Cisplatin	Organoids from cisplatin-sensitive and -resistant human OC tissue samples	Cisplatin resistance closely correlates with cell senescence and glucose metabolism in OC organoids High Aurora-A expression induces OC chemoresistance, and correlates with poor survival in OC patients Aurora-A knockdown reduces OC progression and sensitizes OC cells response to cisplatin in vivo	[61]
Multiple agents	Patient-derived ovarian and endometrial cancer organoids	Combination of carboplatin and paclitaxel results in a notable decrease in viability Bevacizumab as a single agent showed a modest impact on cell viability Combination of cisplatin with paclitaxel had similar impacts on cell viability	[50]
Paclitaxel	Primary tumor organoid cell lines from seven unique OC patients	Upregulated ABCB1 expression in paclitaxel-resistant TOV-21G, OVCAR3 and novel ovarian tumor organoid models Synergistic effects of poziotinib or lapatinib (by direct inhibition of paclitaxel-induced ABCB1 expression) with paclitaxel in resistant TOV-21G and OVCAR3 cells	[58]
Palladium (II)-η^3^-allyl 4c complex bearing N-trifluoromethyl N-heterocyclic carbenes	Patient-derived OC organoids	Nucleophilic attack on the η^3^-allyl fragment High efficacy in OC organoids and low liver toxicity	[79]
Platinum-based chemotherapy	Patient-derived organoids from 47 patients with adenocarcinoma of the ovary or uterus	Correlation between platinum resistance in gynecologic cancer and metabolic signatures Clinical outcome prediction following carboplatin plus paclitaxel chemotherapy by metabolic signatures	[56]
Platinum-based chemotherapy	Fifty-six organoid lines from thirty-two patients, representing all main subtypes of OC	HGSOC organoids were sensitive to platinum-based treatments Low-grade serous OC, mucinous and borderline tumor organoids were more resistant to platinum-based chemotherapy Enhanced resistance of organoids derived from recurrent OC to platinum-based chemotherapy	[26]
Platinum-based chemotherapy	Seven human HGSOC organoids	Extracellular matrix signaling alteration in HGSOC cells by matrix stiffness and platinum chemotherapy HGSOC cell protection against cisplatin-induced apoptosis via focal adhesion kinase (FAK) and YAP signaling COL6 confers relapse HGSOC patient cells with cisplatin induced adhesion and cisplatin resistance	[60]
Platinum drugs, Paclitaxel, Olaparib	Patient-derived OC organoids	Similar copy number variations among organoids and primary tumors Higher sensitivity of the BRCA1-mutated organoid to olaparib and platinum drugs Resistance of the clear cell OC organoid to platinum drugs, paclitaxel and olaparib	[33]

**Table 4:** The use of organoids for disease modeling and targeted therapy sensitivity testing.

Therapeutic Agent	Organoids	Main Results	References
**Human-derived organoids**			
Anti-PD1 therapy	Short-term human HGSOC organoids	Stromal infiltrating mast cells are associated with HGSOC progression and immunoevasive microenvironment Patient stratification according to stromal infiltrating mast cells predicts immune checkpoint blockade therapy effectiveness and prognosis in HGSOC	[73]
Anti-PD-1/PD-L1 antibody	Human HGSOC organoid/immune cell co-cultures	HGSOC organoid/immune cell co-cultures resemble the immune microenvironment of OC Anti-PD-1/PD-L1 antibody downregulates BRD1 expression in immune cells BRD1 inhibition enhances NK cell activation and tumor cell killing	[74]
Apatinib	Patient-derived HGSOC organoids	High FBN1 levels in cisplatin-resistant OC organoids and tissues FBN1 regulates glycolysis and angiogenesis via the VEGFR2/STAT2 pathway FBN1 knockdown downregulates tumor progression and increases cisplatin sensitivity in OC in vivo	[62]
Artesunate	Patient-derived OC organoids	Artesunate shows antiproliferative activity and induces G1 arrest in OC Synergistic effects of Artesunate with carboplatin and paclitaxel	[78]
AZD5153	Patient-derived OC organoids	AZD5153 reverses palbociclib resistance in vitro by targeting cell cycle-related proteins and the MAPK/PI3K-AKT pathway AZD5153-palbociclib synergy inhibits OC growth and induces apoptosis in vitro and in vivo	[77]
BET/PI3K-AKT-mTOR inhibitors	Patient-derived clear cell OC organoids	BET/PIK3-AKT pathway inhibitors synergy in clear cell OC CPI0610 and PI3K-AKT inhibitors synergize to induce p53-independent apoptosis	[80]
Birinapant	Organoid panel of 7 epithelial OC cell lines and 10 platinum- resistant primary patient OC samples	Birinapant-carboplatin combination treatment promotes apoptosis in platinum resistant OC	[65]
Carboplatin, PARP inhibitor, CHK1 inhibitor, ATR inhibitor	Short-term patient- derived HGSOC organoids	Tumor mutational status correlates with organoid functional testing Organoid functional profiling is associated with DNA repair mutation and gene copy number analysis Combination of prexasertib with carboplatin or gemcitabine promotes fork instability	[30]
CWP232291	Organoids from cisplatin-sensitive and cisplatin-resistant patients	Inhibition of organoids’ growth	[59]
Erlotinib-ABT-737-combination	Patient-derived serous OC organoids	microRNA-3622b-5p sensitizes OC chemo-resistant cells to platinum, and represses OC migration and invasion microRNA-3622b-5p inhibits both Bcl-xL and EGFR-mediated BIM induction Synergistic effects of the erlotinib-ABT-737-combination in OC organoids	[81]
Two hundred and forty kinase inhibitors	Four patient-derived tumor organoids established from two ovarian and one peritoneal high-grade serous carcinomas and one carcinosarcoma of the ovary	Highly tumor-specific responses with little overlap among inhibitors BGT226 showed activity in all tumors	[52]
Multiple agents	Organoid culture from HGSOC malignant effusions	APR-246, CB-5083, MK-1775 and Sorafenib showed the most consistent inhibitory effects	[43]
Multiple agents	Patient-derived serous OC organoids	Culture-dependent drug-induced growth arrest and cytostatic drug efficacy in OC cells More diverse and of lower therapeutic potential drug responses in organoids Homologous recombination deficiency scores are associated with drug effects in organoids	[51]
Multiple agents	Thirty-six whole-genome- characterized organoids from twenty-three OC patients	Organoid drug response is associated with patients’ clinical response. Interpatient drug response heterogeneity associated with genetic makeup	[53]
Naftopidil	Patient-derived HGSOC organoids	Naftopidil inhibits OC cell proliferation Naftopidil promotes the expression of Bim, Puma and Noxa proteins Naftopidil-induced BH3-only members sensitizes OC cells to ABT-737 and Trametinib treatments, in vitro and ex vivo and in organoids	[75]
NPB, PARPi	Recurrent human epithelial OC organoids	Reduced cell survival of OC after combined treatment with NPB and PARPi	[68]
PARPi Platinum-based chemotherapy	Primary and metastatic OC organoids including all histological subtypes (PARPi untreated or post-PARPi treatment recurrent OC)	Organoids’ drug response correlates well with clinical response Organoids can be used as a tool to predict PARPi sensitivity and evaluate therapeutic schemes to overcome it	[69]
PEITC, PARP inhibitor	Patient-derived HGSOC organoids	H2O2 treatment increases HGSOC cell sensitivity to BMN 673 PEITC treatment induces excessive ROS levels PEITC/PARP-inhibitor synergy inhibits growth of OC spheroids and patient-derived organoids	[67]
Proteasome/ HDAC inhibitor combination	Ovarian-carcinosarcoma-patient-derived organoid	Rare mutation in TP53 resulting in the deletion of N131 Bortezomib and belinostat combination as the most effective therapeutic regimen	[70]
Proteasome/ HDAC inhibitor combination	Patient-derived ovarian and endometrial cancer organoids	Sensitivity of most ovarian and endometrial cancer organoids and cell lines to combination treatment with a proteasome and HDAC inhibitor Autophagic flux elevation in resistant cells via ixazomib and romidepsin Autophagy inhibition sensitizes resistant cells to ixazomib and romidepsin in vitro, by suppressing HDAC-6 activity	[71]
PXD101	Primary OC clinical specimens grown in three-dimensional organoid culture	PXD101, alone or in combination with other chemotherapeutics, displays an in vitro growth-inhibitory activity in OC cell lines, clinical samples and xenograft models PXD101 enhances the acetylation of A-tubulin induced by docetaxel and the phosphorylation of histone H2AX induced by carboplatin	[72]
ReACp53	Human OVCAR3 organoids	ReACp53-carboplatin synergy in targeting a subset of human OC cells in vitro. Enhancement of HGSOC cell targeting upon addition of ReACp53 to carboplatin	[63]
ReACp53	Human HGSOC organoids	ReACp53 promotes apoptosis, cell cycle arrest and p53 degradation ReACp53 affects cell viability and proliferation of mutant p53 cancer cells in organoids Transcriptional reactivation of p53 by ReACp53	[64]
Stichoposide C	Patient-derived HGSOC and endometrioid OC organoids	Stichoposide C downregulated proliferation, promoted cell cycle arrest, and induced apoptosis and autophagy via the inhibition of the AKT/mTOR signaling pathway in OC cells	[76]
UBE2N	Patient-derived HGSOC organoids	UBE2N inhibitor sensitized patient-derived organoids to carboplatin	[66]
**Mouse-derived organoids**			
Gemcitabine, g-MDSCs, anti-PD-L1 antibodies	Murine fallopian-tube-epithelium-derived organoid-based platform	Durable complete responses in *Tp53*^−/−^; *Ccne1*^OE^; *Akt2*^OE^; *Kras*^OE^ HGSOC Tumor genotype-specific therapeutic efficacy	[45]

## Data Availability

Not applicable.
